# Evaluation of dimensional accuracy, flexural strength and surface roughness of 3D-printable denture base resin modified with different concentrations of cerium oxide nanoparticles: a comparative in-vitro study

**DOI:** 10.1186/s12903-025-05840-7

**Published:** 2025-04-15

**Authors:** Ibrahim A. Mohamed, Mohamed sherine El Attar, Sonia M. El Shabrawy, Eman Zaghloul Alrafah

**Affiliations:** 1 Department of Prosthodontics, Faculty of Dentistry, University of Alexandria, Alexandria, 21525 Egypt; 2 Department of Dental Biomaterials, Faculty of Dentistry, University of Alexandria, Alexandria, Egypt

**Keywords:** Cerium oxide, Nanoparticles, 3D-printed, Denture base resin, Dimensional accuracy, Flexural strength, Surface roughness

## Abstract

**Background:**

Low antimicrobial activity is a major drawback of three-dimensional (3D) printed denture bases, so the incorporation of antimicrobial nanoparticles possesses an effective antifungal and antibacterial effect. However, it is important to assess the outcome of adding such nanofillers on the dimensional accuracy, flexural strength, and surface roughness of 3D-printed denture bases. This in vitro study aimed to evaluate dimensional accuracy, flexural strength, elastic modulus, and surface roughness of 3D printed denture base resin modified with different concentrations of cerium oxide nanoparticles as an antimicrobial agent.

**Methods:**

A total sample of (*N* = 72) was 3D printed as Cerium oxide particles were mixed with the denture base resin to acquire these groups: Group I (control) with no nanoparticles (*N* = 24), Group II with 0.5 wt.% cerium oxide nanoparticles (*N* = 24), and Group III with 1 wt.% cerium oxide nanoparticles (*N* = 24). The printed samples (*N* = 72) were tested for printing accuracy by a digital caliper, and flexural strength (*n* = 12) with a universal testing machine, while Surface roughness (*n* = 12) was assessed by a profilometer. For data analysis, One and 2-Way ANOVA, followed by Tukey post hoc, and the Kruskal Wallis test followed by the Dunn post hoc test were used with Bonferroni correction (*P* ≤ .05).

**Results:**

Regarding printing accuracy specimens there was a statistically significant deviation between the control group and the 1% cerium oxide group in length and width percent error (%). there was no significant effect on flexural strength in all the groups. The 1% Cerium Oxide group recorded the highest mean values. There was a significant difference among all groups of surface roughness before polishing; the control group had the highest mean values. After polishing there was no significant effect.

**Conclusions:**

Regarding printing dimensional accuracy, the 0.5% cerium oxide group had no significant deviation in the printed specimens, while the 1% group had a significant deviation regarding the length and width dimensions of the specimens. The addition of cerium oxide led to a slight improvement in the flexural strength and elastic modulus of the 3D-printed resin without a significant amount. The polishing process of the modified specimens is required to enhance the surface roughness of the material.

## Background

PMMA photocurable resins are being introduced as new biomaterials for the 3D printing technology where adding nanomaterials into the resin enhances their mechanical and biological properties [1].

Low antimicrobial activity is a major drawback that can be improved by modifying the components of the material, changing the technique of polymerization, and adding antimicrobial or reinforcement materials [2, 3].

Nanoparticle addition has a strong effect on organic polymers of the materials that change the physical, chemical, and mechanical properties. Their diameter ranges from 1–100 nanometres which is a small size with a large surface area and unique properties [4, 5].

Commonly used nanoparticles include silver (Ag), titanium dioxide (TiO_2_), silicon dioxide (SiO_2_), and aluminum oxide (Al_2_O_3_). Another nanoparticle material with a considerable antimicrobial effect is zirconium oxide (ZrO_2_), with increasing the filler content of ZrO_2_nanoparticles (>1wt%) the antimicrobial activity and surface roughness significantly increased but it decreased after three months of aging [6, 7].

Cerium oxide (CeO_2_) nanoparticles have unique surface properties, good stability, and biocompatibility so it used in industries such as biomedical, and technological applications, and surface protection against oxidation or UV irradiation [8–10]. In the dental field, cerium oxide nanoparticles are combined with hydroxyapatite coatings to enhance osseointegration and decrease inflammatory reactions and microbial growth for orthopedic implants [11].

Moreover, it can significantly reduce graft rejection rates. Incorporating different percentages of cerium oxide nanoparticles into 3D printed denture polymers can improve the mechanical properties such as impact strength and bending resistance of these denture bases [12, 13].

The study aimed to analyze the effect of incorporating nano-cerium oxide powder into 3D-printed denture base resin on the dimensional accuracy regarding length, width, and thickness. Moreover, analyzed the effect of incorporating nano-cerium oxide powder into 3D-printed denture base resin on flexural strength and elastic modulus for all test groups. As well as the evaluation of the surface roughness percentage reduction of both unpolished and polished sides of the same 3D-printed specimens.

The null hypothesis of this study was that there would be no significant difference in incorporating nano-cerium oxide powder into 3D-printed denture base resin on the dimensional accuracy regarding length, width, and thickness. Moreover, there would be no significant difference in incorporating nano-cerium oxide powder into 3D-printed denture base resin on flexural strength, and elastic modulus for all test groups. As well as there would be no significant difference in surface roughness percentage reduction of both unpolished and polished sides of the same 3D-printed specimens.

## Methods

A total sample size of 72 samples (24 specimens per group) was determined for the study following Rosner’s method [14] assessed by G*Power 3.1.9.7. assuming a 5% alpha error and 80% study power.

**Fig. 1:**
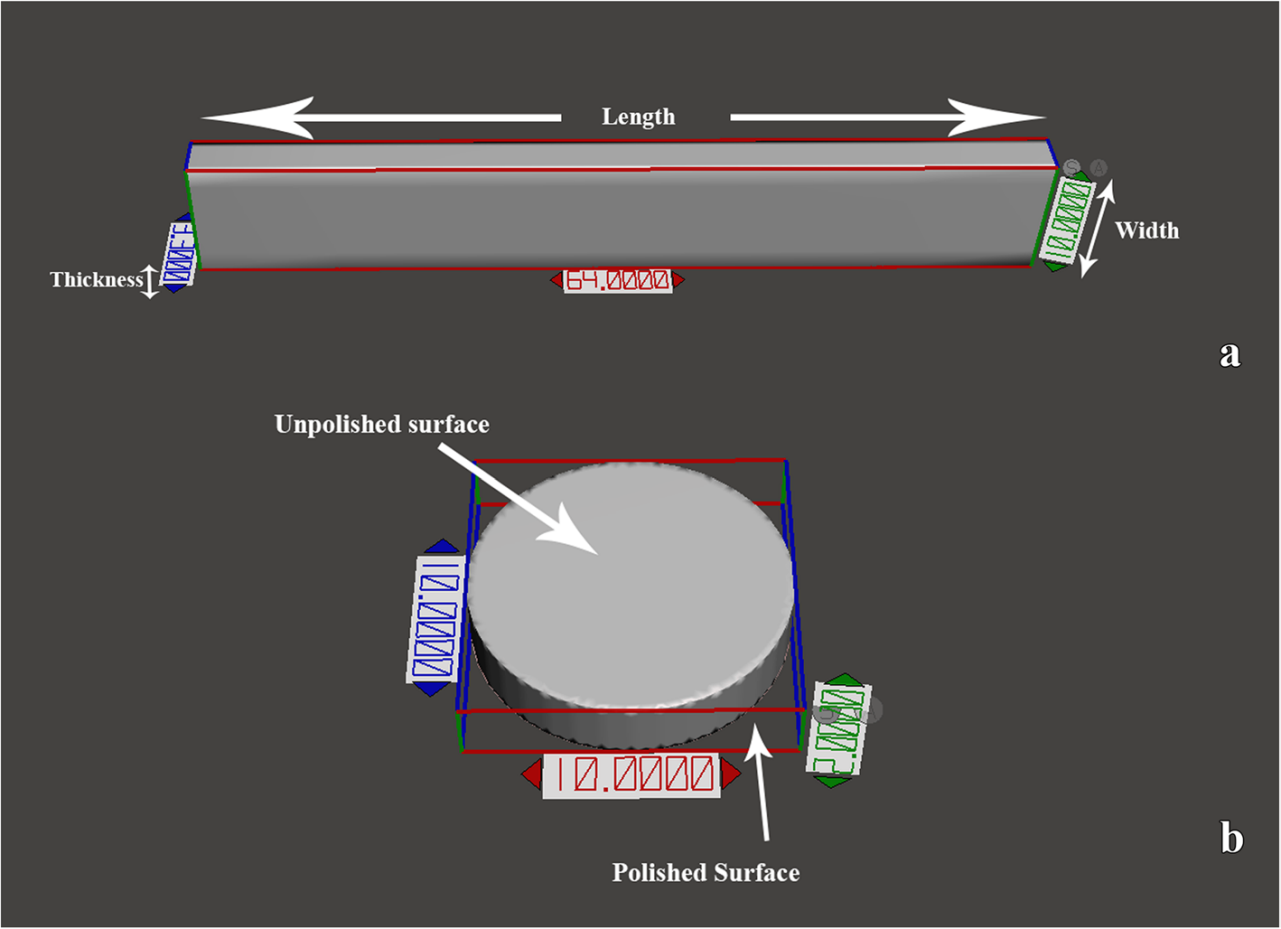
3-Dimensional designs of test specimens. a. specimen for flexure strength test and dimensional accuracy test with dimensions of 64 × 10x3.3 mm. b. disc-shaped specimen of the surface roughness test with dimensions of 10 × 2 mm

The specimens were designed and fabricated using CAD software (Meshmixer 3.5 (Autodesk Inc)). The roughness samples (Fig. 1b) were designed to form 10 × 2 mm discs (*n* = 12) [15]. The flexure test samples (*n* = 12) were designed following the ISO 20795–1:2013 specifications with dimensions of 64 × 10x3.3 mm (Fig. 1a) [16].

Dimensional accuracy was assessed using the flexure specimens before fracture (Fig. 1a) according to the formula A = ((MV-RV)/RV) × 100 by comparing the measurements of each sample to the STL file dimensions. Where “A’’ is the accuracy, “RV’’ is the virtual dimensions, and “MV’’ is the specimen-measured dimensions [17].

**Fig. 2:**
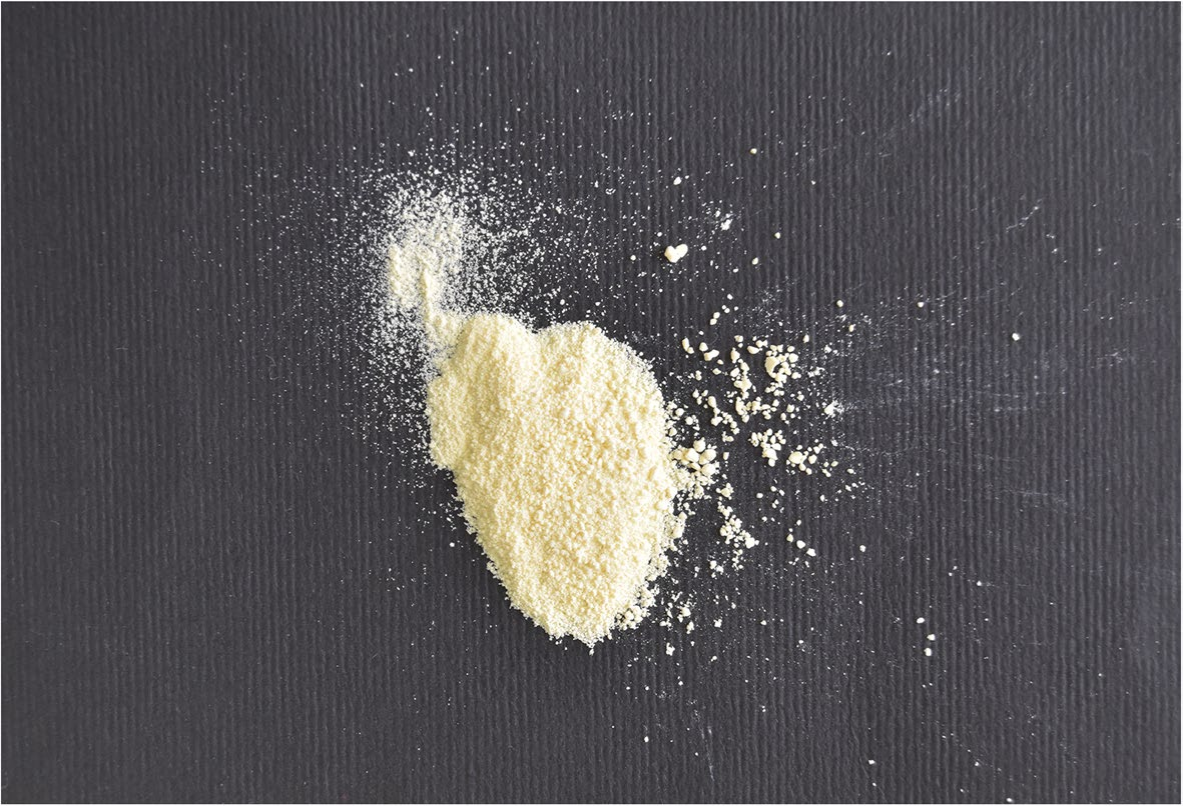
Cerium oxide nanoparticles

Cerium oxide nanoparticles (Spheroidal shape- less than 100 nm; Nano Gate Co) (Fig. 2) were weighted using a digital balance (AS 220-R2; RADWAG® Wagi Electroniczne) to obtain the required quantity of particles. Then, the cerium oxide nanoparticles were mixed with 70% ethanol for 20 min with a magnetic stirrer (F91T; Falc) to improve the textural properties of the nanoparticles, particularly surface area, and increase the phase purity by ethanol washing [18], then the ethanol was evaporated on the hot plate stirrer and muffle furnace (HD-150 “PA”; Hobersal) at 80°c to obtain dry nanoparticles [19].

The liquid photo-curable denture resin (Dentute base; IFun) and cerium oxide nanoparticles were mixed in glass beakers according to the required filler percentages using a glass rod, then stirred by using a magnetic stirrer for 1 h at 500 rpm, followed by ultrasonication (T-14; L & R manufacturer) at 40 W for 30 min then stirred again with a magnetic stirrer for 2 h before printing to obtain three groups: 0.5 wt.% cerium oxide modified resin, 1wt.% cerium oxide modified resin and a control group of non-modified resin [20].

**Fig. 3:**
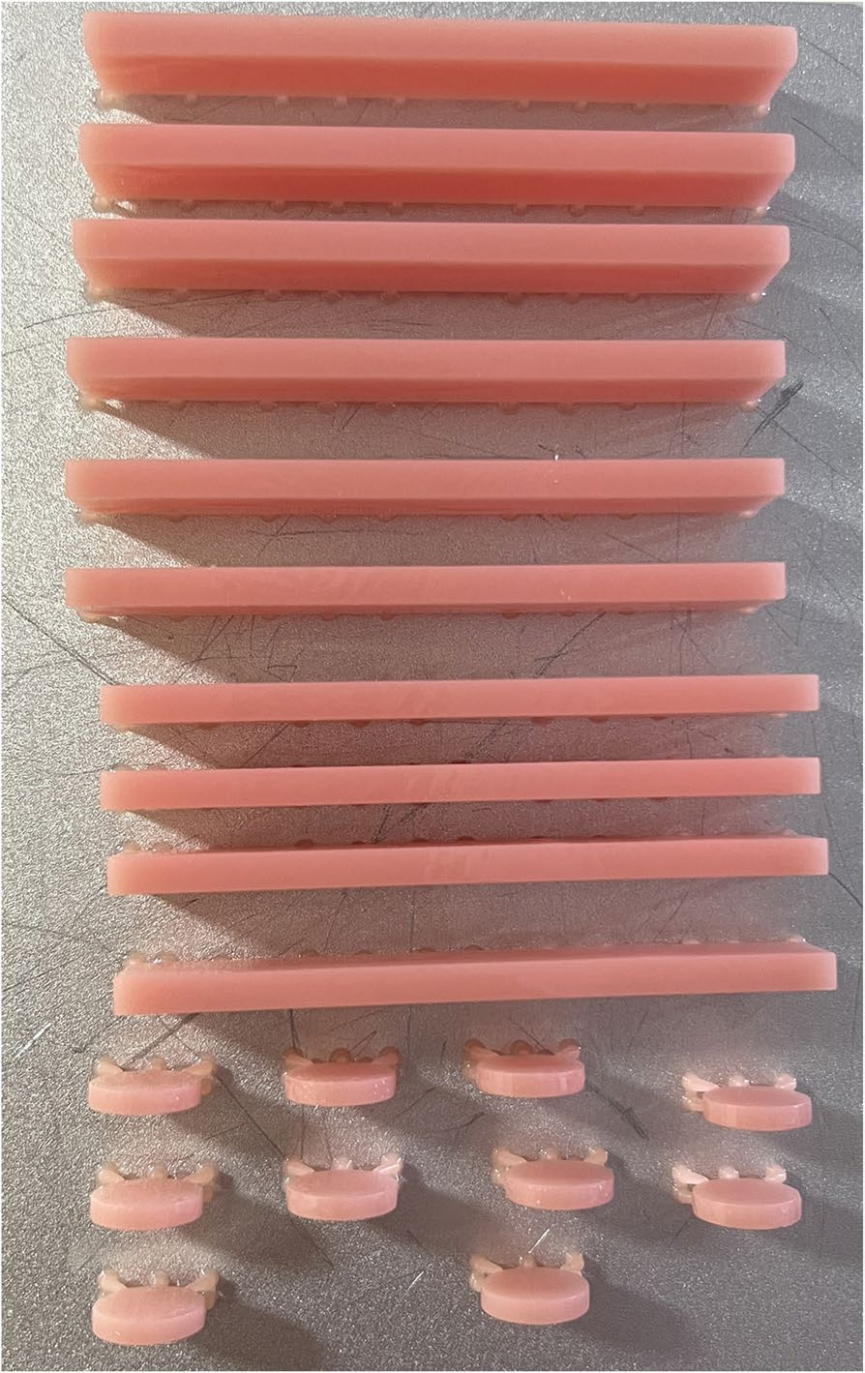
Denture base resin specimens modified with 1wt% cerium oxide nanoparticles

The nanoparticle content must be adequately low, not above 1wt%, to confirm better content dispersion regarding Go et al. [19], who evaluated the antifungal effect of cerium oxide nanofillers addition on 3D printed denture base resin with different concentrations (0-control, 0.5, 1.0, 2.0, and 4.0wt%) on Candida albicans and concluded that cerium oxide nanoparticles had an antifungal effect when it added to 3D printed denture base resins. And its antimicrobial effect increased with high concentration. In this study, the two percentages 0.5wt% and 1wt% were chosen as a previous study concluded that the more the cerium oxide nanoparticles content, the more the filler aggregation [10]. Then, the specimens were obtained using a 3D printer (EPAX; X1-4KS) (Fig. 3). After printing, samples were ultrasonicated in isopropyl alcohol for 3 min to remove excess monomer and post-cured in a post-curing machine (Mogassam) for 3 min to achieve complete polymerization [15].

The cross sections of fractured resin samples were analysed by SEM (scanning electron microscope) with an original magnification of (× 25 000) after the three-point bending test to assess the homogeneity of the mixture and the potential agglomeration of nanoparticles.

The dimensional accuracy was tested using the flexure test specimens then followed by the Flexural strength test for the same specimens (Fig. 1a). A digital caliper (± 0.01 mm) (HARDENED) was used to measure the width, length, and thickness of each flexure specimen at three points (one in the center and two at a 1-mm distance from each sample edge) [21]. All dimensions were measured three times by the same investigator and analyzed with the average value. The percentage of average error was obtained by comparing dimensions with the virtual CAD software measurement [22].

**Fig. 4:**
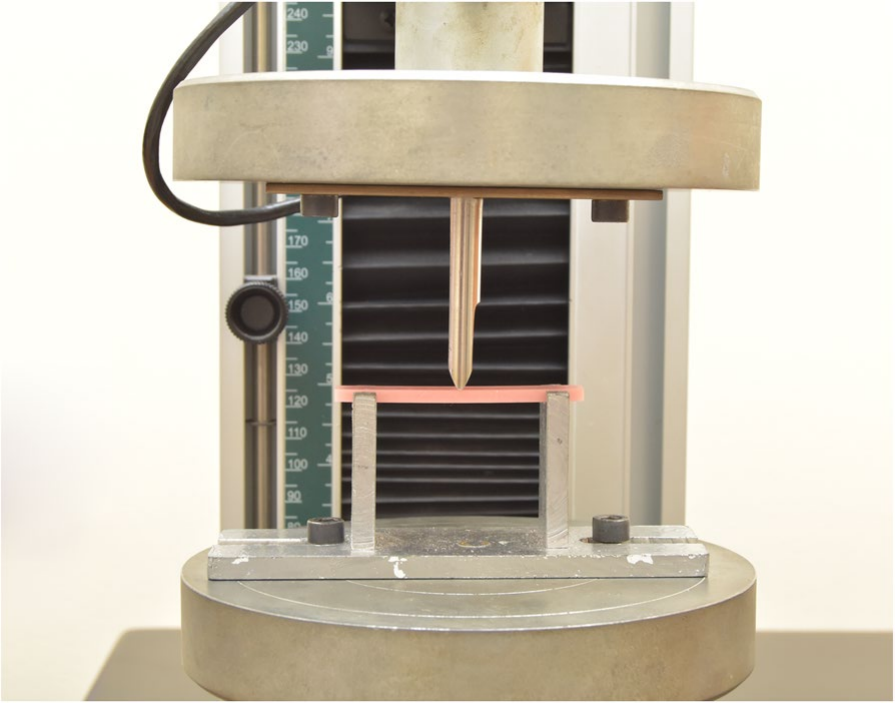
Denture base resin specimens on the UTM (universal testing machine) machine

**Fig. 5:**
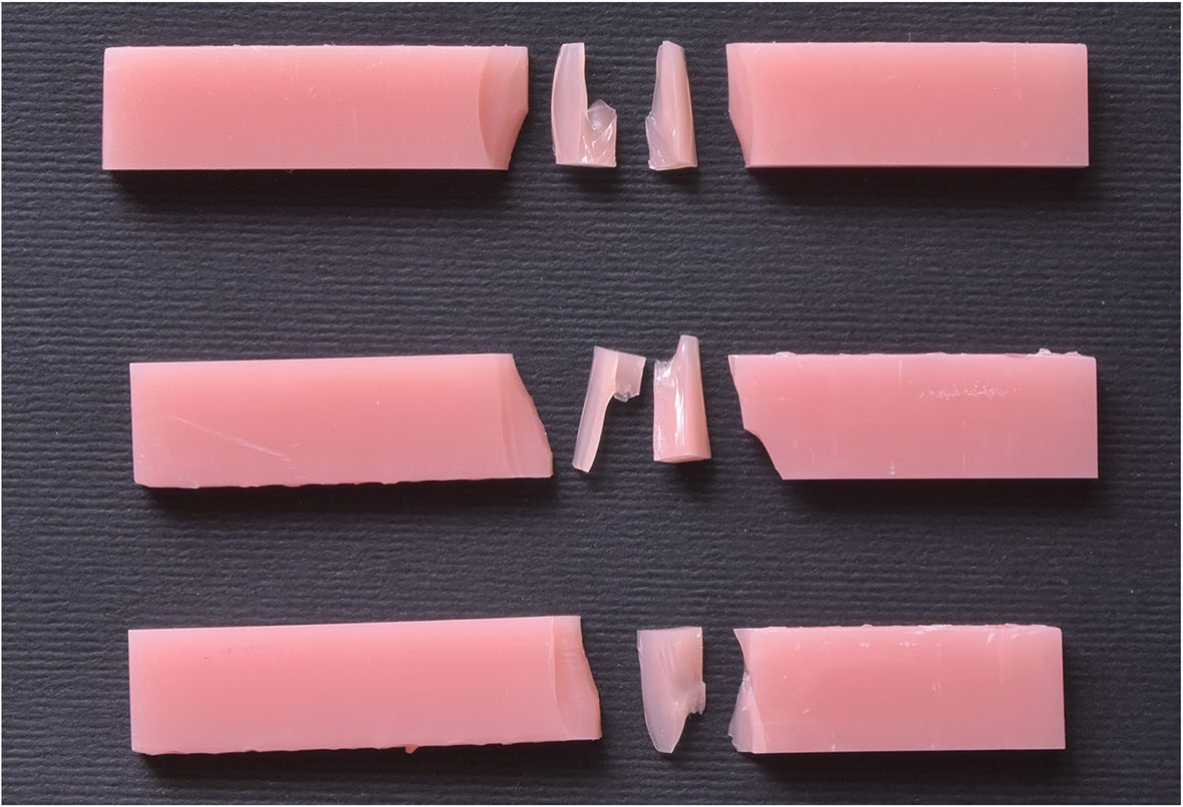
Flexural strength specimens after fracture

Flexural samples were stored in distilled water before testing for 24 h. Then, the Flexural strength was measured with a three-point bending test. Each sample was mounted on a universal testing machine (UTM) (5ST; Tinius Olsen) (Fig. 4) and the load was applied to the sample midpoint at a crosshead speed of 5 mm/min until fracture (Fig. 5). The Flexural strength in MPa was measured using an equation FS = 3FL/ 2bh^2^. where F is the ultimate force in N, L is the support span length (50 mm), and b and h are the specimen width and thickness in millimeters [23].

After calculating Flexural strength, the modulus of elasticity was obtained by the formula E = (P/d) (L^3^/ [4bh^3^]). Where E is the modulus of elasticity in MPa, P is the load, d, and L are displacement and support span length in millimeters, while b and h are the specimen width and thickness in millimeters [24].

Disc-shaped samples were stored in distilled water before testing for 24 h [15]. One surface of each sample was finished using an acrylic stone bur (Medium grit PC2; Acrylic polishing kit; Shofu Co) for 2 min and silicon carbide papers of grit 1500 and 2000 each for 30 s under water cooling at 300 rpm, then polished using a rag wheel and pumice slurry in water (Steribim super; BEGO GmbH & Co KG) were used for 2 min at 1500 rpm, and the specimens were ultrasonicated for 5 min in distilled water. while the other surface was still unpolished [25, 26]. The polishing process was standardized across all groups and was done by one investigator using the same method applied to the three groups regarding the time and the speed of movement.

**Fig. 6:**
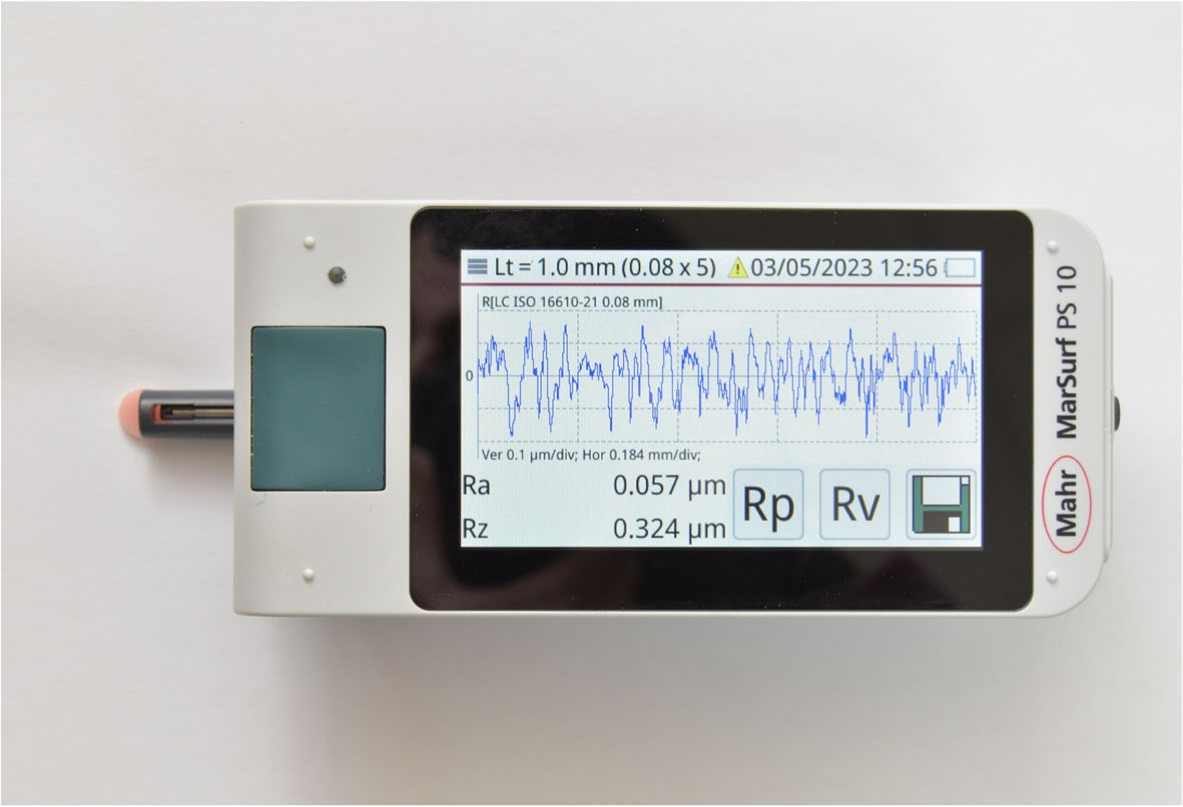
Surface roughness specimens on the contact profilometer machine

The average surface roughness value was assessed for the unpolished and polished surfaces for each sample with a contact profilometer (Marsurf PS 10; Mahr) (Fig. 6) [27].

Data were analyzed using IBM SPSS statistical software (Macintosh, v.28.0; IBM Corp). One-way ANOVA was used to assess the difference in Flexural strength, elastic modulus, and length percent error. 2-way ANOVA was used to assess the effect of nanoparticle percentage and polishing on surface roughness. The ANOVA tests were followed by Tukey post hoc followed by Bonferroni correction. Kruskal Wallis test followed by Dunn post hoc was used to compare percent change in Ra, width, and thickness percent error. All tests were 2-tailed (*p* ≤ 0.05 for all tests). The sample size was estimated assuming 5% alpha error and 80% study power. The mean (SD) flexural strength values of 3D printed denture bases at different nanoparticle concentrations were 76.7 (11.2) at 0%, 93.6 (11) at 0.5%, and 96.4 (15.8) at 1% (18). Based on differences between independent means using the F test and the highest SD = 15.8 to ensure enough study power, a sample of 12 samples per group was required, yielding an effect size = 0.55.

## Results

**Fig. 7:**
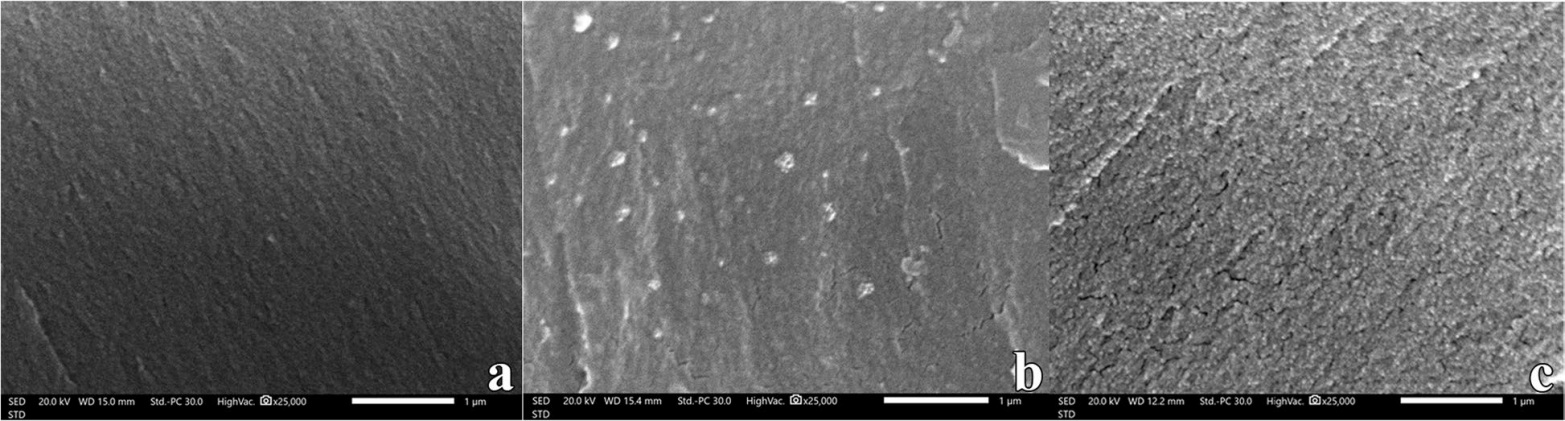
SEM images for cerium oxide nanoparticles dispersion in modified resin groups (original magnification × 25 000). **a **control group without nanoparticles. **b **0.5 wt.% cerium oxide group. **c **1 wt.% cerium oxide group demonstrated well-distributed filler clusters

The SEM analysis (Fig. 7) displayed that the nanoparticles were observed as scattered and evenly distributed in the specimens of modified 3D-printed resin (0.5 wt. % and 1 wt. %) cerium oxide groups.

For the printing dimensional accuracy, the results showed a statistically significant difference in the length percent error (%) between the control and 1% cerium oxide (*p*_*2*_ < 0.0001*), and between the 0.5% and 1% cerium oxide groups (*p*_3_ = 0.001*). However, no significant difference between the control and 0.5% cerium oxide groups was observed. The highest length percent error was recorded by the 1% cerium oxide group (−1.04 ± 0.15%) followed by the 0.5% cerium oxide group (−0.92 ± 0.14%). The least printing length percent error was displayed by the control group (−0.84 ± 0.16%) (Table 1).

**Table 1:** Comparison of length, width, and thickness percent error (%) between the study groups

		Control (*n*=12)	0.5% Cerium Oxide (*n*=12)	1% Cerium Oxide (*n*=12)	*p* value
**Length**	Mean ± SD	−0.84 ± 0.16	−0.92 ± 0.14	−1.04 ± 0.15	<0.0001*
***Pairwise comparison***		*p*_*1*_=0.061, *p*_*2*_<0.0001*, *p*_3_= 0.001*			
**Width**	Median (IQR)	0.40 (1.20)	0.15 (0.75)	−0.30 (0.90)	<0.0001*
	Min - Max	−1.10 – 2.90	−1.50 – 2.30	−1.30 – 1.60	
***Pairwise comparison***		*p*_*1*_=0.168, *p*_*2*_<0.0001*, *p*_3_= 0.018*			
**Thickness**	Median (IQR)	−1.21 (3.26)	−1.52 (3.26)	−0.61 (2.58)	0.084
	Min - Max	−7.27 – 4.24	−6.67 – 6.67	−3.64 – 2.12	

*Statistically significant difference at *p* value≤0.05. *p*_1_: Comparison between control and 0.5% Cerium Oxide, *p*_2_: Comparison between control and 1% Cerium Oxide, *p*_3_: Comparison between 0.5% Cerium Oxide and 1% Cerium Oxide

Regarding the width percent error (%), the results showed a statistically significant difference between the control and 1% cerium oxide (*p*_*2*_ < 0.0001*), and between the 0.5% and 1% cerium oxide groups (*p*_3_ = 0.018*) and the control displayed the highest percent error (0.40 (1.20)) With no significant difference was observed between the control and 0.5% cerium oxide groups (Table 1).

Regarding the specimens’ thickness percent error (%). There was no statistically significant difference between the control group, the 0.5% group, and the 1% group. The 1% cerium oxide displayed the least printing percent error (−0.61 (2.58)), and the 0.5% cerium oxide group recorded (−1.52 (3.26)) while the control displayed the highest percent error (−1.21 (3.26)) (Table 1).

For flexural strength, the 1% Cerium Oxide group recorded the highest mean values (64.06 ± 4.29 MPa) followed by the 0.5% Cerium Oxide (63.55 ± 3.87 MPa). The least flexural strength values were recorded by the control (62.14 ± 7.66 Mpa) (Table 2) With no significant difference observed between the control group, 0.5% group, and the 1% group.

**Table 2:** Comparison of flexural strength (MPa) between the study groups

		Control (*n*=12)	0.5% Cerium Oxide (*n*=12)	1% Cerium Oxide (*n*=12)
**Flexural strength**	Mean ± SD	62.14 ± 7.66	63.55 ± 3.87	64.06 ± 4.29
	95% CI	57.27, 67.01	61.09, 66.00	61.33. 66.79
F test		0.385		
(*p* value)		(0.683)		

For the modulus of elasticity, there was no significant difference observed between the control group, the 0.5% group, and the 1% group. The highest modulus values were measured by the 1% Cerium Oxide (2192.01 ± 325.20 Mpa) followed by the 0.5% Cerium Oxide (2053.57 ± 778.45 Mpa). The lowest mean value was measured by the control group (1886.09 ± 744.46 Mpa). (Table 3).

**Table 3:** Comparison of elastic modulus (MPa) between the study groups

		Control (*n*=12)	0.5% Cerium Oxide (*n*=12)	1% Cerium Oxide (*n*=12)
**Elastic modulus**	Mean ± SD	1886.09 ± 744.46	2053.57 ± 778.45	2192.01 ± 325.20
	95% CI	1413.08, 2359.09	1558.97, 2548.17	1985.39, 2398.63
F test		0.667		
(*p* value)		(0.520)		

**Fig. 8:**
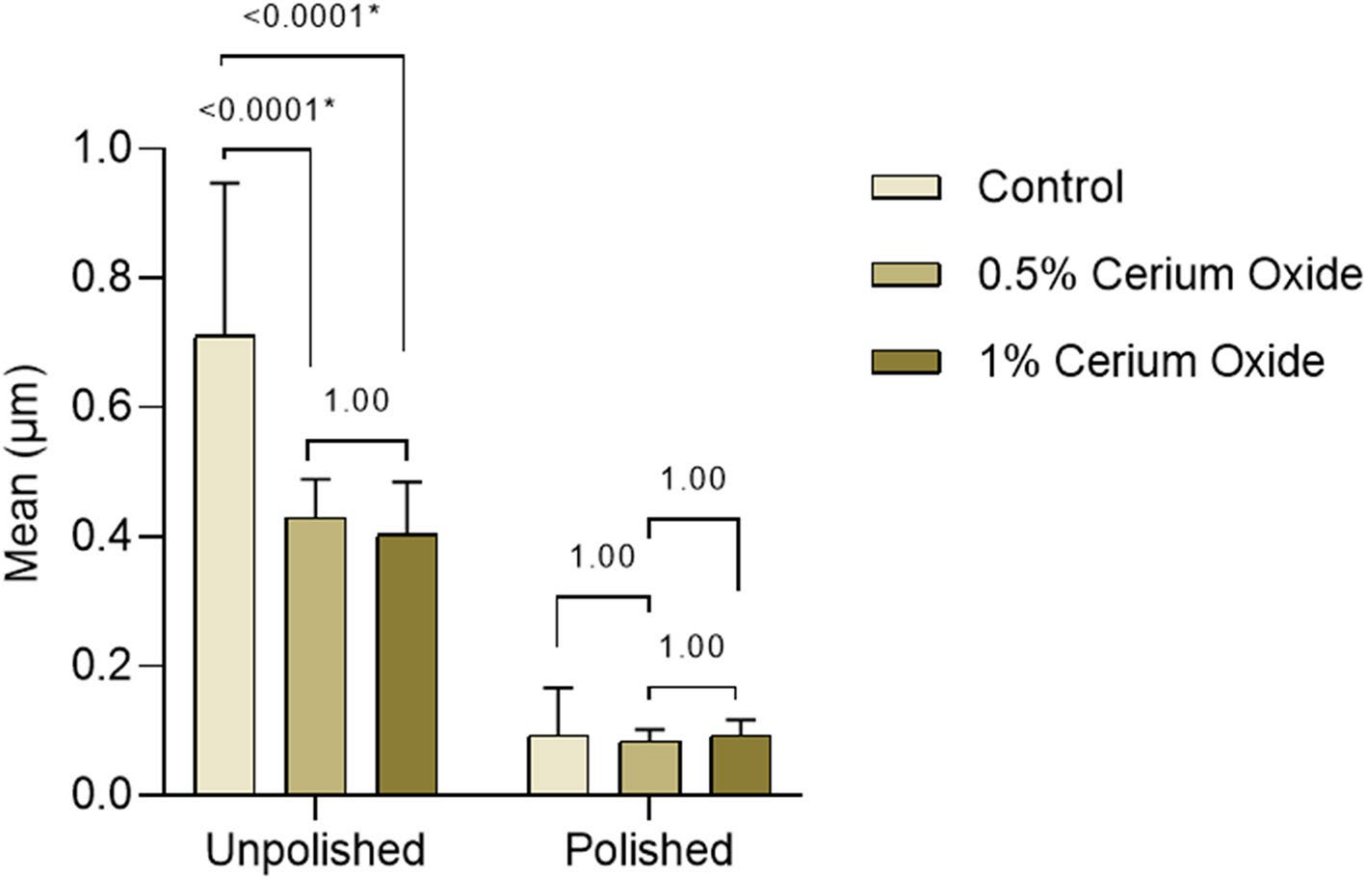
Pairwise comparison between denture resin materials before and after polishing

Regarding the surface roughness of the unpolished specimens, there was a significant difference observed between the control and 0.5% cerium oxide and between the control and 1% cerium oxide groups. However, there was no significant difference between the 0.5% cerium oxide and 1% cerium oxide groups and after polishing there was no significant difference present between the study groups (Fig. 8).

The control group recorded the highest percent reduction (%) in surface roughness among the study groups after polishing by (86.68 ± 6.78%) followed by the 0.5 group by (79.92 ± 4.86%) then the 1% group by (76.26 ± 6.44%) (Table 4).

**Table 4:** Comparison of percent reduction (%) in surface roughness between the study groups after polishing

		Control (*n*=12)	0.5% Cerium Oxide (*n*=12)	1% Cerium Oxide (*n*=12)
% reduction	Mean ± SD	86.68 ± 6.78	79.92 ± 4.86	76.26 ± 6.44
	95% CI	82.37, 90.98	76.82, 83.01	72.17, 80.35
	Median (IQR)	88.78 (7.66)	80.98 (6.95)	75.88 (12.66)
	Min - Max	70.61 – 93.75	70.43 – 86.30	67.14 – 86.90
H test		12.848		
(*p* value)		(0.002*)		
Pairwise comparisons		*p*_*1*_=0.035*, *p*_*2*_=0.002*, *p*_3_= 1.00		

*p*_1_: Comparison between control and 0.5% Cerium Oxide, *p*_2_: Comparison between control and 1% Cerium Oxide, *p*_3_: Comparison between 0.5% Cerium Oxide and 1% Cerium Oxide

The 2-way ANOVA (Table 5) revealed that the filler content showed a significant statistical effect (*P* < 0.0001*, F = 14.798, Ƞp2 = 0.310) on the surface roughness of the study groups. Also, the polishing process had a significant statistical effect (*P* < 0.0001*, F = 267.609, Ƞp2 = 0.802) on the surface roughness values. Moreover, there was an inclusive overall effect between the resin filler content and the polishing process (*P* < 0.0001*, F = 13.998, Ƞp2 = 0.298) with the polishing displayed as the uppermost effect (Ƞp2 = 0.802).

**Table 5:** Two Way ANOVA assessing the effect of denture base resin and polishing process on surface roughness

	df	Mean square	F test	*p* value	Ƞp^2^
Filler content	2	0.179	14.798	< 0.0001*	0.310
Polishing process	1	3.235	267.609	< 0.0001*	0.802
Filler content x polishing process	2	0.169	13.998	< 0.0001*	0.298
Corrected model	5	0.786	65.040	< 0.0001*	0.831

^*^Statistically significant difference at* p* value ≤ 0.05. Ƞp^2^: Partial Eta Squared, Model Adjusted R Squared = 0.819

## Discussion

In this in vitro study, different concentrations of cerium oxide nanoparticles have been introduced to 3D printed denture base resin as an antimicrobial agent with unique surface properties and biocompatibility that decreases inflammatory reactions [8, 11]. Therefore, the aim of the study was to evaluate different parameters such as dimensional accuracy regarding length, width, and thickness, moreover analyze the effect of incorporating nano-cerium oxide powder into 3D-printed denture base resin on flexural strength, and elastic modulus for all test groups. As well as the evaluation of the surface roughness percentage reduction of both unpolished and polished sides of the same 3D-printed specimens.

The liquid photo-curable denture resin and cerium oxide nanoparticles (0.5 wt.% and 1 wt.%) were stirred by using a magnetic stirrer for 1 h followed by ultrasonication for 30 min, then stirred again by using a magnetic stirrer for 2 h before printing.

In this study, the two percentages 0.5wt% and 1wt% were chosen as the previous research by GO et al., [19] concluded that the more the cerium oxide nanoparticles content (with 2 wt.% and 4 wt.%), the more the filler aggregation, therefore the time of magnetic stirring was increased by one hour more than Chen et al., [20] moreover, the printing of the specimens was done immediately after magnetic stirring to confirm a proper dispersion of the cerium oxide nanoparticles into the 3D-printed modified resin and decrease the potential for agglomeration of the nanoparticles.

The SEM analysis was performed to assess the homogeneity of the incorporated nanoparticles into the modified resin and ensure that there was no agglomeration of the cerium oxide nanoparticles. The nanoparticles were observed as scattered and evenly distributed in the specimens of modified 3D-printed resin (0.5 wt.% and 1 wt.%) cerium oxide groups (Fig. 7).

In the current study, the results showed that there was no statistically significant difference in comparing the Flexural strength and elastic modulus between the control group, the 0.5% and the 1% group. Therefore, the null hypothesis of both was accepted.

On the other hand, there was a statistically significant difference in the dimensional accuracy of printed specimens regarding the length and width, also, there was a statistically significant difference in surface roughness of the unpolished sides of the same 3D-printed specimens between the control group, the 0.5% and the 1% group. Therefore, the null hypothesis of both was rejected.

The dimensional accuracy affects the adaptation and fitment of the denture base to the mucosa, and the attachment precision and fitting of the implant-retained prosthesis, which consistently impair retention, mechanical properties, longevity, and maintenance of the prosthesis, which leads to patient dissatisfaction [28, 29].

Since the dimensional accuracy of dental prosthesis has an impact on denture prosthesis retention and durability, different factors should be considered, including the quantity and arrangement of supporting structures. As well as the building degree, the speed and energy of the polymerizing source, the placement of 3D specimen on the build platform, the number of layers, material shrinkage, and post-curing measures [30, 31].

In the current study, regarding the dimensional accuracy of the printed specimens, on comparing the control group with the two test groups, all specimens showed a percent error. However, the length and width of the 1% cerium oxide group specimens showed a significant deviation after printing on being compared to the control group. while the 0.5% showed no significant difference compared to the control group.

This may be due to the printing orientation used as it was in a zero-degree position and the platform moved in a vertical direction on the tank while the samples were formed by growing in width while exposing the monomer to light in a vertical direction, but the length is exposed to the lateral resolution of the laser light. This may be a limiting factor preventing more accurate printing of the length dimension [32]. Also, the length of the specimen is the largest dimension that was affected more by the minimal shrinkage during the processing as well as the post-curing process [22, 32].

Regarding the deviation in the width of the 1% cerium oxide group, this may be due to the peel force while the vertical movement of the specimens inside the liquid resin in the tank, with more layer build-up and increased bonding between the layers while printing [21, 22].

In the present study, these findings of the dimensional accuracy of the length and the width of the specimens on being printed with a zero-degree tilt were in agreement with Shim et al., [28] and Al-Qarni et al. [22], who concluded that Specimens printed at 0 degrees had an error rate associated with specimen length, followed by specimen width, then thickness.

Moreover, on comparing the 0.5% group and the 1% group, there was a significant deviation regarding the length and width of the specimens, the amount of deviation was increased with the higher percent amount of the cerium oxide nanoparticles. This was discussed that the agglomeration of cerium oxide nanoparticles with high filler content may affect the cross-linking between the resin matrix, which causes a poor adhesion between the nanofiller and the polymer matrix [33].

In the study, the difference in the cerium oxide nanoparticle concentrations caused different percent changes in dimensional accuracy, which were in agreement with Reyes et al., [10] who stated similar results. Those findings were explained by Tahayeri et al., [32] who stated that during the printing process, the color of the resin affects the intensity of the laser as the darker materials require more intensity of the laser to cure a higher depth than the lighter ones.

Regarding that, The increase in the cerium oxide nanoparticle concentrations may decrease the amount of laser light that reaches the resin material during the printing process, which may be one of the causes of increased printing inaccuracy with the increased amount of cerium oxide nanoparticles regarding the length and width of 0.5% and 1% cerium oxide groups [10].

The denture's Flexural strength determines how well a denture base material can tolerate mastication stresses. In the present study, the Flexural strength was tested using the 3-point bending test, which simulates the stresses applied to the denture in use [34].

In the current study, regarding the Flexural strength and the modulus of elasticity, there was no statistically significant difference between the control group and the 0.5% group as well as the 1% group. The use of the 1wt% Cerium Oxide group recorded the highest mean values, followed by the 0.5wt% Cerium Oxide, while the least flexural strength values were recorded by the control group.

This may be due to the presence of cerium oxide nanoparticles, which exerted an effect on the resin chains’ segment mobility. The crystal size and crystallinity of CeO2 nanofillers also might cause a decrease in creep displacement with increasing the cerium oxide nanoparticles amount, so the addition of rigid cerium oxide nanoparticles improved the creep resistance, which enhanced the flexural strength of the modified resin with cerium oxide nanoparticles than the control group [10, 35].

These results were agreed with the results of GO et al., [19] who conducted a non-significant difference regarding the Flexural strength and elastic modulus between the 0.5 and 1wt% nanoceria groups and the control. However, the flexure strength of the 2.0 wt.% and 4.0 wt.% groups showed significantly lower values than the control group (*P* > 0.05). as well as agreed with AlGhamdi et al., [36] who concluded that the flexural strength was increased by the addition of TiO2 nanoparticles to 3D-printable denture base resin.

The surface roughness (Ra) of denture base materials is affected by the properties of the material, polishing techniques, and the dental hygiene habits of the patients. It plays a key role in plaque accumulation and bacterial adhesion, which leads to staining and influences the patient's comfort and aesthetics [37].

Denture bases with rough surfaces usually manifest microbial adherence as well as denture stomatitis [38]. Therefore, finishing and polishing the denture base material is a must to reduce plaque accumulation and achieve an optimum surface roughness [39].

In the current study, the printed specimens were finished using an acrylic bur and silicon carbide paper, then polished using a rag wheel and pumice slurry, while the other surface was still unpolished to compare the average surface roughness value for both unpolished and polished surfaces for each sample with a contact profilometer. The finishing and polishing process was standardized across all groups and was done by one investigator using the same method applied to the three groups regarding the time and speed of the movement.

Regarding the unpolished surface of the specimens, it showed a statistically significant reduction in surface roughness of 3D printed denture base resin as a result of the addition of cerium oxide nanoparticles, but it exceeded the 0.2-μm acceptable Ra limit for denture bases based on AL‐HARBI et al., and MOHAMED et al., that would increase the incidence of plaque accumulation which leads to microorganism colonization and denture stomatitis. [26, 40].

This surface roughness reduction may be due to the appropriate dispersion of cerium oxide nanoparticles within the resin material and filling the inner spaces between the chains of polymers, leading to a smoother surface (Fig. 7).

These results agreed with Zidan et al., [41] who conducted that the incorporation of nanoparticles could close the micro gaps between the resin chains, leading to minimizing the irregularities and the voids of the surface of the samples during processing. And also in agreement with Mhaibes et al. [42], who stated the same results by evaluating the influence of the addition of titanium oxide nanotubes on the surface properties of 3D-printed denture base materials and concluded that the surface roughness of the denture base resin decreased with the addition of 1.0 and 1.5 wt.% TiO_2_ nanotubes.

Regarding the polished surface of the specimens, there was no statistically significant difference between the study groups however the control group recorded the highest percent reduction (%) in surface roughness followed by the 0.5% and 1% groups.

The present study evaluated the effect of different concentrations of cerium oxide that manifested an influence on the mechanical properties of the 3D printed resin for denture base material, where some limitations were found including a larger sample size as well as the evaluation of the incorporation of different concentrations of cerium oxide nanoparticle on being printed with different orientation mechanisms such as 45 and 90 degrees.

## Conclusions

The study’s findings led to the following conclusions:

1. Regarding the dimensional accuracy, (the length and width) of the printed specimens using a zero-degree tilt showed that the 0.5 wt.% cerium oxide group was more dimensionally stable while the 1 wt.% group had a significant deviation.
2. The addition of cerium oxide to the 3D-printed resin for the (0.5 wt.% and 1 wt.%) group showed a slight improvement in flexural strength and elastic modulus of the modified 3D-printed specimens.
3. In both the control and the modified test groups (0.5 wt.% and 1 wt.%) the polishing process of the specimens enhanced the surface roughness of the material which significantly would impact the reduction of microbial adhesion on the dental prosthesis which correspondingly would reduce the formation of denture stomatitis on long term use.
4. Further research could be conducted to evaluate the effect of higher concentrations of CeO₂ nanoparticles with different printing orientations and long-term clinical performance on the modified 3D-printed specimens.

## Data Availability

The datasets used and/or analyzed during the current study are available from the corresponding author on reasonable request.

## Abbreviations

PMMA: Poly methyl Metha acrylate
3D: Three dimensional
UV: Ultraviolet
Ag: Silver
TiO_2_: Titanium dioxide
SiO_2_: Silicon dioxide
Al_2_O_3_: Aluminum oxide
ZrO_2_: Zirconium oxide
CeO_2_: Cerium oxide
CAD: Computer-aided design
UTM: Universal testing machine
SEM: Scanning electron microscope
